# Brain insulin signaling and cerebrovascular disease in human postmortem brain

**DOI:** 10.1186/s40478-021-01176-9

**Published:** 2021-04-15

**Authors:** Zoe Arvanitakis, Ana W. Capuano, Hoau-Yan Wang, Julie A. Schneider, Alifiya Kapasi, David A. Bennett, Rexford S. Ahima, Steven E. Arnold

**Affiliations:** 1 Rush Alzheimer’s Disease Center, Rush University Medical Center, 1750 W. Harrison Street, Suite 1000, Chicago, IL 60612 USA; 2 Department of Molecular, Cellular and Biomedical Science, City University of New York School of Medicine, 160 Convent Avenue, New York, NY 10031 USA; 3 Department of Biology, Neuroscience Program, Graduate School of The City University of New York, 365 Fifth Avenue, New York, NY 10061 USA; 4 Division of Endocrinology, Diabetes and Metabolism, Johns Hopkins University School of Medicine, 333 East Monument Street, Baltimore, MD 21205 USA; 5 Department of Neurology and the Massachusetts Alzheimer’s Disease Research Center, Massachusetts General Hospital, Harvard Medical School, 149 13th Street, Charlestown, MA 02129 USA

**Keywords:** Diabetes, Insulin, Brain, Infarct, Atherosclerosis, Amyloid angiopathy

## Abstract

Insulin is an important hormone for brain function, and alterations in insulin metabolism may be associated with neuropathology. We examined associations of molecular markers of brain insulin signaling with cerebrovascular disease. Participants were enrolled in the Religious Orders Study (ROS), an ongoing epidemiologic community-based, clinical-pathologic study of aging from across the United States. Using cross-sectional analyses, we studied a subset of ROS: 150 persons with or without diabetes, matched 1:1 by sex on age-at-death and education. We used ELISA, immunohistochemistry, and ex vivo stimulation with insulin, to document insulin signaling in postmortem midfrontal gyrus cortex tissue. Postmortem neuropathologic data identified cerebrovascular disease including brain infarcts, classified by number (as none for the reference; one; and more than one), size (gross and microscopic infarcts), and brain region/location (cortical and subcortical). Cerebral vessel pathologies were assessed, including severity of atherosclerosis, arteriolosclerosis, and amyloid angiopathy. In separate regression analyses, greater AKT1 phosphorylation at T^308^ following ex vivo stimulation with insulin (OR = 1.916; estimate = 0.650; *p* = 0.007) and greater pS^616^IRS1 immunolabeling in neuronal cytoplasm (OR = 1.610; estimate = 0.476; *p* = 0.013), were each associated with a higher number of brain infarcts. Secondary analyses showed consistent results for gross infarcts and microinfarcts separately, but no other association including by infarct location (cortical or subcortical). AKT S^473^ phosphorylation following insulin stimulation was associated with less amyloid angiopathy severity, but not with other vessel pathology including atherosclerosis and arteriolosclerosis. In summary, insulin resistance in the human brain, even among persons without diabetes, is associated with cerebrovascular disease and especially infarcts. The underlying pathophysiologic mechanisms need further elucidation. Because brain infarcts are known to be associated with lower cognitive function and dementia, these data are relevant to better understanding the link between brain metabolism and brain function.

## Introduction

Diabetes affects more than 34 million persons in the US, with the older age groups being the most impacted [11]. Indeed, about 40% of persons over the age of 60 years have a diagnosis of diabetes, and this same age group has the highest number of medical complications including in the brain [11]. Insulin resistance is an early and characteristic feature of the most common types of diabetes, and specifically type 2 diabetes [32] and has been extensively studied in adipose, muscle, and other tissues. Less is known about insulin in the brain, or how insulin resistance relates to neuropathology, the underlying substrate of brain dysfunction. Much of the current scientific knowledge in brain is derived from animal studies, and recent data are characterizing forms of insulin resistance which may differentially associate with particular neuropathologies of dementia [23, 37]. While these and other approaches contribute importantly to advancing science, research on insulin signaling and resistance in human brain tissue is needed to better understand uniquely human conditions such as Alzheimer’s disease (AD) dementia. Yet, such data in persons with or without diabetes are sparse. In a recent pathologic study, alterations in expression of insulin receptor signaling pathway genes in human brain were found to be modulated by diabetes medications [17]. In a recent study from our group, we found that brain insulin resistance measures from persons matched on diabetes status, were related to AD pathology and lower cognitive function [8].

Diabetes causes vascular complications in many organs. The relation of insulin and related factors with cardiovascular disease is increasingly studied, including for the insulin receptor substrate (IRS) and downstream molecules such as AKT as they relate to myocardial infarction and cardiac vessel pathologies such as atherosclerosis [14]. While the brain is a less well studied organ in this regard, given that diabetes is an established risk factor for stroke, examining the relation of insulin signaling in humans with the underlying neuropathology of stroke promises to advance the field of diabetes, insulin resistance, cerebrovascular disease, and brain dysfunction [15]. Indeed, animal studies have associated the brain insulin signaling pathway, including for IRS1 and AKT, to hypoxic brain injury [31]. In humans, several neuroimaging studies have shown that diabetes and peripheral insulin resistance (as assessed in blood biomarkers) are related to brain infarcts [19]. Moreover, genetic and epigenetic data have found that specific genes and gene polymorphisms, including of the IRS1 gene, are associated with stroke [29]. Few published data are available on the relation of brain insulin signaling and resistance in persons with and without diabetes, to cerebrovascular pathology.

Here, we examined the association of human brain insulin resistance, as assessed by molecular signaling measures including IRS1 and serine/threonine-protein kinase [AKT], with pathologically-defined cerebrovascular disease. We used data and brain tissue from 150 elderly, deceased and autopsied persons who were with or without diabetes, who participated in the Rush Religious Orders Study (ROS), a prospective, community-based, clinical-pathologic study of aging with a high autopsy rate. Biochemical, immunohistochemical, and ex vivo brain stimulation with insulin measures, were collected in postmortem midfrontal gyrus cortex, as previously described in this group of 150 subjects [8]. We examined for associations of the brain insulin signaling measures with cerebrovascular pathology, and specifically with brain infarcts (including by size and location) and cerebral vessel pathologies including atherosclerosis, arteriolosclerosis, and amyloid angiopathy. We also examined whether diabetes affected associations of the insulin measures with cerebrovascular pathology.

## Materials and methods

### Cohort, clinical and pathologic data, and case selection

Subjects were enrolled in the Religious Orders Study (ROS), which is a prospective, community-based clinical-pathologic cohort study of aging, started in 1993 and ongoing across the US, with follow-up and autopsy rates exceeding 90% [9]. The Rush University Institutional Review Board approved the study, and all subjects signed an informed consent and were asked to sign an anatomical gift act for brain donation at time of death. Subjects underwent annual clinical evaluations, which included a medical history, physical examination, neuropsychological testing, and drug data collection. The presence of diabetes was based on the annually-collected clinical data which documented information based on the medical history, and data on visually-inspected medications (including anti-diabetes medications), as previously published [1, 3, 24]. Other vascular risk factors were also documented, including a history of hypertension assessed on an annual basis. Also, apolipoprotein E ε4 *(APOE ε4)* genotype data were collected.

At the time of death, a brain autopsy was performed (locally where the participant died within the US) using a uniform and standardized procedure, with a short postmortem interval (PMI; mean < 10 h). Rapid brain autopsies are performed to maintain good tissue preservation. In addition, we perform snap freezing of fresh postmortem tissue using brass metal plates at − 80 °C. The average PMI of tissue samples in this study was 6.8 h (SD = 4.9) and the range was 1.0–26.5 h. While it is possible that a long PMI may affect postmortem protein degradation and the brain insulin signaling parameters, immunostaining profiles for most proteins extracted from brain tissue remain unchanged even after a PMI of longer than 50 h. Neuropathologic data were then systematically collected at the laboratory of the Rush Alzheimer’s Disease Center (RADC, senior neuropathologist and author JAS). In brief, tissues from one hemisphere were frozen (at − 80 °C) and from the other, were fixed in paraformaldehyde [25]. Neuropathologic assessments were conducted, blinded to clinical data, as previously described [25]. A uniform gross and histologic evaluation examined for common age-related neuropathologies including cerebrovascular disease and AD pathology [4, 5, 7, 25]. Briefly, gross (macroscopic) infarcts were identified on gross examination and classified by number, volume (in mm^2^) and location [25]. Each gross infarct was then dissected and confirmed on microscopic examination using H&E, and classified by age (chronic, subacute, acute). Microinfarcts are not visible to the naked eye by definition, and were identified under microscopy in blocks of nine brain regions that were paraffin-embedded and stained with H&E [5]. Location and age of microinfarcts were also recorded. In analyses, only chronic infarcts were considered.

Cerebral vessel pathology data were systematically collected, as previously published [7]. Atherosclerosis severity was graded by visual inspection of vessels in the Circle of Willis, using a semi-quantitative scale, based on involvement of each artery and number of arteries involved, from 0 (no atherosclerosis) to 6 (severe atherosclerosis, with all visualized large arteries affected or one artery completely occluded). Arteriolosclerosis severity was documented on the histologic examination, using H&E stained sections of the anterior basal ganglia, and graded based on vessel wall thickening, and ranged from 0 (no arteriolosclerosis) to 7 (complete small vessel occlusion). Amyloid angiopathy severity was graded in several neocortical brain regions, based on degree of immunohistochemical labeling with anti-amyloid-*β*, as previously published [6, 36]. In analyses for this study, the severity of each of the vessel pathologies was grouped into three levels as described in the methods below.

For this study, among all ROS participants who died and came to autopsy, we selected 150 cases with completed neuropathologic data collection and brain tissues available for this study, using rigorously applied inclusion and exclusion criteria, as recently described elsewhere [8]. In order to obtain a spectrum of potential insulin resistance and allow for examination of differential effects by diabetes status, we selected 75 subjects with and 75 without diabetes, matched by sex (woman: woman and man: man), and balanced by group on age at death and education. Among the 75 pairs, we identified 39 pairs (39 with and 39 without diabetes) with PMI ≤ 12 h, for the e*x vivo* stimulation experiments, as described elsewhere [38].

### Human brain insulin signaling and related measures

Brain data described below, were collected in one of three established, academic laboratories headed by experts in their respective fields (authors RSA, SEA, and H-YW). We used an ELISA kit to assess for levels of proteins in the signaling pathway in previously frozen middle frontal gyrus cortex (MFC) tissues from the 150 selected subjects from the ROS with and without diabetes [8]. After thawing, tissue was homogenized in lysis buffer and supernatant aliquots were assayed in duplicate using the PathScan^®^ ELISA kit, a solid phase sandwich ELISA (Cell Signaling, Danvers, MA): measurements obtained were on the total IRS1, catalog #7328; pIRS1 (S307), catalog #7287; AKT1, catalog #7170; and pAKT1 (Thr308), catalog #7252.

We also used immunohistochemical methods to obtain data on the presence, quantitation, and localization of insulin signaling in brain tissue, in paraformaldehyde-fixed, paraffin-embedded blocks of MFC from the same 150 subjects, as previously described [8, 30]. Tissue was processed and methods applied as detailed elsewhere, using antibody against pS^616^IRS (Invitrogen 44-550G, Rabbit 1:500), and automated microscopy digital image capture (Image-Pro Plus software, Media Cybernetics), and computer-assisted image analysis to obtain a semi-quantitative variable of number of pS^616^ IRS1 positive neuronal cells/mm^2^ of tissue. In addition, we also measured the optical density (OD) of the immunohistochemical staining within neurons as a more sensitive measure of pS^616^IRS1 protein expression.

In the subset of 78 subjects (39 pairs with and without diabetes; all with PMI ≤ 12 h), we conducted additional data collection in MFC, of insulin-induced IR activation, using the ex vivo stimulation with insulin method described elsewhere [8, 30, 38]: (a) IRS1 recruitment to IRβ, (b) phosphorylation of IRβ at tyrosines 1150/1151 (pY^1150/1151^ IRβ) and (c) phosphorylation of IRβ at tyrosine 960 (pY^960^ IRβ). The values for each of these indicators was derived by the ratio of measure after incubation with 1 nM insulin (IN) or Kreb's Ringer (KR), normalized to total IRβ immunoprecipitated by anti-IRβ antibodies, as previously published [8, 38]. Further, two indicators of insulin-induced AKT1 activation were measured: (a) AKT1 phosphorylation at serine 473 (pS^473^AKT1) and (b) AKT1 phosphorylation at threonine 308 (pT^308^AKT1). The values for these indicators were derived by ratio of the measure after incubation in 1 nM insulin (IN) or Kreb's Ringer (KR), normalized to total AKT1 immunoprecipitated by anti-AKT1 antibodies [8, 38]. Details of the methods used were recently published elsewhere, using a small sample of subjects without diabetes [38]. The methods of the ex vivo stimulation experiments in human tissue have been established and extensively described in our earlier studies, including in tissue from the ROS [30, 38]. The important biological insights derived from the ex vivo stimulation method include the ability to compare the responsiveness of tissue from persons with and without disease, to diverse stimuli such as insulin (or combinations of hormones). The impact of the PMI has been addressed in our earlier publications using postmortem human brains [30] and rat brain [38] with a range of PMIs (from many to few hours). Both studies consistently indicate that brain tissues with PMI ≤ 12 h are suitable for ex vivo stimulation in response to insulin, adiponectin, and leptin. The impact of tissue pH within the PMIs of ≤ 12 h did not significantly influence the tissue responsiveness with comparable dose–response curves. In this study cohort, brain tissues were obtained from age- and sex-matched subjects.

Descriptive data including the medians and interquartile ranges, and graphical display, of the ELISA, immunohistochemical, and ex vivo stimulation measures among the 150 subjects in the current study, is provided in a recent publication including in the published Table 2 and Figs. 1 and 2 [8]. The insulin signaling and related measures were not statistically different when comparing subjects with and without diabetes, as reported [8].

### Statistical analyses

All pathology data (brain infarcts and cerebral vessel pathologies) were ordinal. The number of ordinal levels used in analyses for this study depended on the sparsity of the data especially at the end of the ordinal distribution, so that we could conservatively apply cumulative ordinal models. Brain infarcts were examined as two levels (presence of one or more infarcts, versus absence) with exception of the variable for the presence of “any infarct” that could be examined as three levels (none, single, and multiple). For vessel outcomes including atherosclerosis, arteriolosclerosis, and amyloid angiopathy, it was possible to examine three levels (graded as none or minimal severity (reference group); mild severity; and moderate or severe).

We used separate logistic regressions to examine the associations between each insulin markers and binary/two-level pathologies and we used cumulative ordinal models to examine three level ordinal pathologies. Models adjusted for age-at-death, and sex. Age was centered at the mean for interpretation purposes. We employed standard diagnostic methods and graphical examination of residuals to verify that assumptions underlying the statistical models were adequately met. We have three sets of brain insulin signaling predictors that were tested. The first set included the z-score of the ELISA-based insulin markers (2 variables): the ratio of pS^307^IRS1over total IRS1, and the ratio of pT^308^AKT1 to total AKT1. The second set included the z-score of the immunohistochemistry-based insulin marker (1 variable) represented by the number of pS^616^ IRS1 staining cells per mm^2^ of tissue. And the third set included the z-scores of the ex vivo insulin stimulation markers (5 variables): IRS1 recruitment to IRβ, pY^1150/1151^IRβ, pY^960^IRβ, pS^473^AKT1, and pT^308^AKT1. We applied Bonferroni adjusted alpha (α) for each set of variables tested, respectively set 1 to 3 used the adjusted alpha 0.025 (0.05/2), 0.05, and 0.01 (0.05/5). All analyses were conducted using SAS/STAT software, Version 9.4 of the SAS^®^ system for Linux.

## Results

### Sample characteristics

Table 1 shows the demographic and neuropathologic characteristics of the 150 subjects included in the study. In the total group, the mean age at death was 86.6 years, and half were women. Half the group had evidence for the presence of any brain infarct, and multiple infarcts in particular. A third of the group had a moderate to severe grade of vessel pathologies including atherosclerosis, arteriolosclerosis, and amyloid angiopathy. The demographic characteristics including age, sex, and education did not differ between persons with and without diabetes, by study design and as reported elsewhere [8]. We examined the association between insulin signaling markers and history of hypertension (identified at any time point in the study). We did not find any associations (data not shown).

**Table 1:** Characteristics of subjects by diabetes status

	Total	Diabetes	No Diabetes
	n = 150	n = 75	n = 75
Demographic			
Age-at-death in years, mean (SD)	86.6 (6.1)	86.6 (5.9)	86.7 (6.3)
Women, n (%)	72 (48%)	36 (48%)	36 (48%)
Education in years, mean (SD)	18.1 (3.3)	18.2 (3.1)	18.1 (3.4)
*APOE ε4,* n (%)	36 (24%)	15 (20%)	21 (28%)
*Neuropathologic*			
Brain infarcts (presence), n (%)			
Any type infarct (by size and location)			
One infarct	29 (19%)	13 (17%)	16 (21%)
More than one infarct	46 (31%)	32 (43%)	14 (19%)
Gross infarcts			
One infarct	28 (19%)	14 (19%)	14 (19%)
More than one infarct	28 (19%)	19 (25%)	9 (12%)
Microinfarcts			
One infarct	26 (17%)	16 (21%)	10 (13%)
More than one infarct	19 (14%)	12 (16%)	7 (9%)
Cortical infarcts			
One infarct	20 (13%)	12 (16%)	8 (11%)
More than one infarct	15 (11%)	9 (12%)	6 (8%)
Subcortical infarcts			
One infarct	28 (19%)	16 (21%)	12 (16%)
More than one infarct	30 (20%)	22 (29%)	8 (11%)
Cerebral vessel pathologies			
Atherosclerosis			
Mild	71 (47%)	31 (41%)	40 (53%)
Moderate	49 (33%)	25 (33%)	24 (32%)
Severe	12 (8%)	8 (11%)	4 (5%)
Arteriolosclerosis			
Mild	44 (30%)	25 (33%)	19 (25%)
Moderate	31 (21%)	12 (16%)	19 (25%)
Severe	15 (10%)	12 (16%)	3 (4%)
Cerebral amyloid angiopathy			
Mild	39 (27%)	31 (41%)	29 (39%)
Moderate	31 (21%)	14 (19%)	17 (23%)
Severe	15 (10%)	6 (8%)	9 (12%)
Alzheimer’s disease pathology			
Global score	0.6 (0.6)	0.7 (0.6)	0.6 (0.5)

### Brain insulin signaling and brain infarcts

Because diabetes is known to increase the risk of stroke, we first examined the association of brain insulin signaling in persons with and without diabetes with the most common underlying pathology of the clinical syndrome of stroke, brain infarcts. We used ordinal logistic regression with number of brain infarcts as the outcome, categorized as one infarct, more than one infarct, and no infarct as the reference group. All analyses controlled for age at death and sex. As shown in Table 2 and using corrected α values as explained in the Methods, we did not find an association of most of the insulin signaling and related measures, including by both ELISA and all but one ex vivo stimulation measure, with brain infarcts regardless of infarct size or location (results on the left). And specifically in the analysis using immunohistochemistry, we did not find an association between the number of IRS1 immunoreactive cells (IRS1 cells/mm^2^) and infarcts (Table 2). In additional analysis, we also examined a measure of the average optical density (OD) of pS^616^IRS1 within neurons, reflecting expression levels of IRS1 on a per neuron basis, as this may be a more sensitive indicator of neuronal insulin resistance. In this analysis, we did find that greater brain insulin resistance as determined by a higher pS^616^IRS1 immunolabeling in neuronal cytoplasm, was associated with a higher number of brain infarcts (OR = 1.610; estimate = 0.476; *p* = 0.013). In the subgroup of 79 subjects with ex vivo insulin stimulation data available, greater AKT phosphorylation (pT^308^AKT1) was associated with a higher number of brain infarcts (Table 2). We next conducted secondary analyses by using logistic regression models with outcomes of gross and microinfarcts and by locations as cortical and subcortical infarcts. Results with pS^616^IRS1 immunolabeling were consistent for gross infarcts (OR = 1.488; estimate = 0.400; *p* = 0.047) and microinfarcts (OR = 1.679; estimate = 0.518; *p* = 0.012) each considered separately, and for gross cortical and micro-subcortical infarcts specifically (data not shown; sample sizes in some cells were smaller). However, there was no other association of AKT phosphorylation following ex vivo stimulation or any of the other insulin signaling and related measures by ELISA or ex vivo stimulation with gross or microinfarcts outcomes, or with infarcts in cortical or subcortical brain regions (Table 2).

**Table 2:** Association of insulin signaling measures with brain infarcts

	OR, Estimate (SE, p)				
	Any infarct	Size of infarct		Location of infarct	
		Gross infarcts	Microinfarcts	Cortical infarcts	Subcortical infarcts
ELISA					
pS^307^IRS1/total IRS1	0.689, − 0.375 (0.169, 0.027)	0.718, − 0.331 (0.190, 0.081)	0.708, − 0.346 (0.195, 0.076)	0.828, − 0.188 (1.206, 0.361)	0.735, − 0.308 (0.185, 0.096)
pT^308^AKT1/total AKT1	1.282, 0.248 (0.159, 0.120)	1.321, 0.279 (0.176, 0.114)	1.135, 0.127 (0.178, 0.477)	1.207, 0.188 (0.189, 0.320)	1.319, 0.277 (0.174, 0.112)
Immunohistochemistry					
IRS1 cells/mm^2^	1.137, 0.129 (0.160, 0.423)	1.017, 0.017 (0.177, 0.925)	1.371, 0.315 (0.183, 0.085)	1.200, 0.183 (0.181, 0.314)	1.058, 0.056 (0.175, 0.7469)
Ex vivo stimulation					
IRS1 recruitment to IRβ	1. 549, 0.438 (0.230, 0.057)	1.245, 0.219 (0.246, 0.372)	1.434, 0.361 (0.255, 0.158)	1.606, 0.474 (0.279, 0.090)	1.252, 0.225 (0.244, 0.356)
pS^473^AKT1	1.536, 0.429 (0.231, 0.063)	1.453, 0.373 (0.254, 0.142)	1.350, 0.300 (0.259, 0.246)	2.158, 0.769 (0.313, 0.014)	1.204, 0.188 (0.244, 0.446)
pT^308^AKT1	**1.916, 0.650 (0.240, 0.007)**	1.940, 0.663 (0.276, 0.016)	1.426, 0.355 (0.255, 0.164)	1.758, 0.564 (0.290, 0.052)	1.575, 0.454 (0.256, 0.076)
pY^1150/1151^IRβ	1.193, 0.177 (0.225, 0.433)	1.070, 0.068 (0.247, 0.784)	1.074, 0.071 (0.256, 0.781)	1.562, 0.446 (0.297, 0.133)	0.979, − 0.0.1 (0.243, 0.930)
pY^960^IRβ	1.604, 0.473 (0.229, 0.039)	1.319, 0.277 (0.244, 0.256)	1.518, 0.417 (0.259, 0.108)	1.667, 0.511 (0.286, 0.074)	1.397, 0.334 (0.245, 0.173)

Separate age and sex adjusted ordinal logistic regression models, for each categorical infarct outcome measure (see text for methods, including description of 3 level outcome for outcome of “any infarct”, and 2 level outcome for other infarct outcomes); results which reach statistical significance are in bold (see methods for corrections for multiple comparisons)

Next we tested if the effect of insulin signaling measure was differential by diabetes status. For that, logistic and ordinal logistic regression models were next repeated with the addition of the terms for diabetes and for the interaction between each insulin signaling measure and diabetes. We found an interaction of pS^616^IRS1 immunolabeling with diabetes, suggesting that greater immunolabeling in persons without diabetes was associated with a higher number of brain infarcts (estimate = 1.203; *p* = 0.046). Interaction terms were otherwise not statistically significant for any of the other markers with diabetes and any of the infarct outcomes (data not shown), suggesting that the presence of diabetes did not affect the relationship of other measures with brain infarct pathology.

### Brain insulin signaling and cerebral vessel pathologies

To better understand plausible mechanisms linking insulin resistance to brain infarcts, we next examined the relation of insulin signaling and related measures to three common cerebral vessel pathologies in aging. We used a series of 24 separate ordinal logistic models adjusted for age at death and sex, and applied correction for multiple comparisons (see “Materials and methods” section). Table 3 shows the results for the eight predictors (insulin measures) and three outcomes of atherosclerosis, arteriolosclerosis, and amyloid angiopathy. We found that greater AKT phosphorylation (pS^473^AKT1) following ex vivo stimulation with insulin, was associated with less amyloid angiopathy. No other association of brain insulin signaling measures with vessel pathology, including atherosclerosis and arteriolosclerosis, was found (Table 3).

**Table 3:** Association of insulin signaling measures with cerebral vessel pathologies

	OR, Estimate (SE, p)		
	Atherosclerosis	Arteriolosclerosis	Amyloid angiopathy
ELISA			
pS^307^IRS1/total IRS1	0.748, − 0.290 (0.1662, 0.081)	1.073, 0.071 (0.160, 0.658)	0.801, − 1.222 (0.163, 0.174)
pT^308^AKT1/total AKT1	1.041, 0.040 (0.160, 0.803)	1.279, 0.246 (0.157, 0.118)	1.104, 0.099 (0.160, 0.537)
Immunohistochemistry			
IRS1 cells/mm^2^	1.280, 0.246 (0.175, 0.159)	0.794, − 0.231 (0.172, 0.179)	1.354, 0.303 (0.1683, 0.934)
Ex vivo stimulation			
IRS1 recruitment to IRβ	1.365, 0.311 (0.228, 0.171)	1.199, 0.182 (0.213, 0.403)	0.900, − 0.106 (0.229, 0.644)
pS^473^AKT1	1.048, 0.048 (0.223, 0.834)	1.206, 0.187 (0.219, 0.392)	**0.496,** − **0.701 (0.253, 0.006)**
pT^308^AKT1	1.215, 0.1951 (0.220, 0.375)	1.115, 0.109 (0.216, 0.613)	0.643, − 0.442 (0.231, 0.055)
pY^1150/1151^IRβ	1.353, 0.3025 (0.2293, 0.187)	1.295, 0.258 (0.222, 0.245)	0.994, − 0.006 (0.234, 0.979)
pY^960^IRβ	1.117, 0.111 (0.221, 0.615)	1.337, 0.290 (0.218, 0.182)	0.573, − 0.557 (0.242, 0.021)

Separate age and sex adjusted ordinal logistic regression models, for each categorical cerebral vessel pathology outcome measure (see text for methods, including categorizing vessel pathology into 3 ordinal levels); results which reach statistical significance are in bold (see methods for corrections for multiple comparisons)

Next we tested if the effect of the insulin signaling measure was differential by diabetes status. For that, we repeated the analysis with pS^473^AKT1, but with the addition of the terms for diabetes and for the interaction between pS^473^AKT1and diabetes. There was no interaction, suggesting that the presence of diabetes did not affect the relationship of the pS^473^AKT1 with amyloid angiopathy (*p* = 0.392).

## Discussion

In this study of elderly autopsied persons with or without diabetes, we found that a greater pT^308^AKT1 following ex vivo stimulation of human postmortem brain tissue, was associated with more cerebrovascular disease, and specifically brain infarcts. This association was present regardless of diabetes status. Also, we found that one measure of greater insulin resistance, as identified by higher pS^616^IRS1 immunolabeling, was associated with more cerebrovascular disease, specifically infarcts including gross infarcts and microinfarcts separately. Interestingly, the association of higher pS^616^IRS1 immunolabeling with number of brain infarcts was present particularly among persons *without* diabetes. No association was found between other measures of insulin resistance (e.g., levels of proteins by ELISA) with any of the infarct outcomes, including cortical or subcortical infarct outcomes. Other analyses with vessel pathologies as outcomes showed no association of the insulin resistance and related markers with atherosclerosis or arteriolosclerosis, except for pS^473^AKT1 following insulin stimulation being associated with less amyloid angiopathy.

Diabetes is an enormous and growing public health problem with serious medical complications, including being a major risk factor for stroke. However, our understanding at the molecular level of mechanisms linking diabetes to stroke is less well understood, specifically the relation of resistance to insulin, a key characteristic of diabetes, to cerebrovascular disease. Several studies have shown that insulin resistance in the periphery (e.g., as assessed by blood markers, such as the homeostasis model assessment–estimated insulin resistance [HOMA-IR] index) is related to cerebrovascular disease, often identified by neuroimaging [19]. Limited data exist using central (brain) insulin resistance measurements. Some research, particularly in animal models of insulin resistance, have shown that brain insulin signaling plays a role in neuropathology including as relevant to cerebrovascular disease, such as IRS1/AKT in hypoxic brain injury [31, 37]. Yet, we are not aware of prior data using human brain tissue from persons with and without diabetes, which examines the relation of brain insulin signaling or resistance to cerebrovascular disease. In the current study, we found that greater brain insulin resistance by pS^616^IRS1 immunolabeling in the midfrontal gyrus cortex, was associated with a higher number of brain infarcts. Further, this association was observed with both number of gross infarcts and microinfarcts separately, suggesting that infarcts of different sizes are associated with brain insulin resistance. Our prior research among the same group of 150 persons showed trends for some of these same brain insulin signaling measures of being higher among persons with diabetes compared to those without, notably for phosphorylated IRS1 (on ELISA) and AKT1 (by ex vivo tissue stimulation), suggesting more brain insulin resistance in diabetes [8]. In the current analyses where we found the presence of an interaction of a brain measure with diabetes, we showed that the association of higher pS-IRS1 immunolabeling with number of brain infarcts was present particularly among persons *without* diabetes. While much work needs to further examine the relation of peripheral to brain insulin resistance and among persons with and without diabetes [2], this finding, coupled with an association of AKT1 with infarcts regardless of diabetes status and with prior work in this and other cohorts, suggests that brain insulin resistance may occur in persons without diabetes and further, that insulin resistance may relate to neuropathology independently of diabetes [30, 34]. Future research will build on these results, to further characterize mechanisms by which brain insulin resistance and modifications on IRS1 relate to cerebrovascular disease, including by examining other molecules in the insulin signaling pathway, using different methods of assessment and in other brain tissues, and testing different sets of persons [10, 13, 18]. Consideration of the role of insulin signaling on the neurovascular unit, cerebral hemodynamics, perfusion and oxygenation, and other factors will be of interest in future research in this field, along with understanding the link of insulin, cerebrovascular disease, and brain function such as cognition [2, 15, 28].

In this study, we showed in the subset of individuals with ex vivo stimulation data, that a greater phosphorylation of a downstream molecular marker of insulin signaling, pT^308^AKT1 following ex vivo stimulation of brain tissue with insulin, was associated with more brain infarcts. We did not observe associations of AKT1 with size (gross vs microscopic) or location of infarct (cortical vs subcortical). Nonetheless, several lines of previously published data point to roles not only of IRS but also of AKT in tissue infarction, mostly in peripheral tissues in animal studies [21]. For example, a recent study of cardiovascular disease found changes in the IRS1-AKT1 signaling pathway in cardiac tissue in a rat model of insulin resistance [14]. While AKT phosphorylation is regulated by several upstream factors such as insulin, AKT isoforms may be differentially expressed in tissues including within the brain (hippocampus) [20, 22]. Further, brain AKT signaling has been implicated in a rat model of ischemic stroke, and may be modulated by statins [33]. In our prior work in the same group of 150 persons with and without diabetes, we found that AKT phosphorylation (pT^308^AKT1/total AKT1, based on ELISA) was associated with another common neuropathology of aging, AD neuropathology, including amyloid and tangles [8]. Furthermore in that study, AKT phosphorylation was associated with lower cognitive function on measures of global cognition and individual cognitive domains (e.g., episodic memory) [8]. Thus, the current result of an association of greater pT^308^AKT1 with more brain infarcts adds to the literature in the field of insulin signaling in the aging brain. Our data suggest that brain insulin resistance may be associated with lower cognitive function through at least two pathologic pathways, cerebrovascular as studied here, and AD as previously published [8]. Further research will examine these possibilities, especially as both are already established as the most common underlying pathologies of dementia [so called Mixed Etiology Dementia (MED)] [26]. While we did not find associations of cortical or subcortical infarcts, location of infarcts and other cerebrovascular characteristics in brain insulin resistance, studies using larger groups of persons with and without diabetes are needed to further advance the scientific knowledge in the field of metabolic disturbances in human brain.

To examine the link of brain insulin resistance to cerebrovascular disease, we also studied vessel pathology measures. We found that greater AKT phosphorylation as measured by pS^473^AKT1 following insulin stimulation, was associated with less amyloid angiopathy. We did not observe any other association between the insulin signaling measures (including AKT phosphorylation by ELISA) with vessel pathologies such as atherosclerosis and arteriolosclerosis. Our findings are unexpected for several reasons. First, with the observed association of pT^308^AKT1 and IRS1 with brain infarcts, one plausible mechanism would be through more vessel pathology, yet our data do not support such a pathophysiologic pathway. The differential association of the insulin-induced pS^473^AKT1 and pT^308^AKT1 with amyloid angiopathy and infarcts, respectively indicates there may be divergent roles of the 3-phosphoinositide-dependent kinase1 (PDK1) and mechanistic target of rapamycin (mTOR) downstream insulin signaling pathways in regulating vascular pathologies. Second, we unexpectedly found one association with less vessel pathology, specifically amyloid angiopathy. While no other relations with vessel pathologies were found and we corrected for multiple comparisons, we cannot exclude a chance finding. Another consideration is that as of yet unknown biological factors may be at play in relating pS^473^AKT1 with less amyloid angiopathy. For instance, biologic compensatory mechanisms in brain may result in less pathology early on in a disease process [16]. Also, pS^473^AKT1 appears to have a complex relation with amyloid including in the cerebrovascular endothelial cells, which needs further elucidation in the field of amyloid angiopathy [12, 27, 35]. Intermediary and other steps in the pathway linking brain insulin resistance to infarcts, in persons with and without diabetes, will need to be characterized in future studies.

This study has several strengths. Using a rigorous nested case–control study design, we studied persons with and without diabetes from a larger cohort study with high autopsy rate and matched subjects 1:1 by sex on age-at-death and education. To examine the insulin signaling pathway in human brain, we used three complementary and established approaches including biochemical, immunohistochemical, and ex vivo insulin stimulation measures, with the latter being a powerful experimental tool yielding detailed data on basal and activation levels of several molecules in the signaling cascade investigated [38]. Further, the neuropathologic data were systematically collected, blinded to clinical data, on a range of cerebrovascular measures, including number, size, and location of infarcts, as well as on semi-quantitative measures of severity grading for three common vessel pathologies in aging. On the other hand, several study limitations are present. While we measured many molecules of the signaling pathway and several aspects of cerebrovascular disease, it is possible that specific isoforms and other signaling molecules, or other neuropathologies not studied (e.g., white matter disease) or under-reported (infarct number and volumes are likely underestimated [39]), are important in the link of insulin resistance to neuropathology. Additional data about features of atherosclerosis, such as plaque ulceration and calcification, are not available. While neuropathologic criteria were systematically collected using standard methods, we recognize that more rigorous and reproducible methods are needed for assessing postmortem cerebrovascular pathologies. We do not have data on the etiology of infarcts. Also, there may be regional difference in the brain in signaling, or in how particular aspects of signaling relate to the neuropathology data. Further, our immunohistochemical finding needs to be interpreted with caution, as there is well recognized variability in staining with this method. More research is needed to confirm our result. We used a rigorous study design, and even with a relatively small sample size, we detected large effects of these novel markers such as the pS^473^AKT1. There were, however, some borderline effect sizes (e.g. in the association of pS^307^IRS1/total IRS1 with any infarcts), that could be further examined in other works. Finally, research participants were relatively healthy, community-dwelling Catholic clergy volunteers who agreed to annual clinical evaluations and autopsy at time or death, who may not be representative of the general population.
